# Limited Correlation between SARS-CoV-2 Serologic Assays for Identification of High-Titer COVID-19 Convalescent Plasma Using FDA Thresholds

**DOI:** 10.1128/spectrum.01154-22

**Published:** 2022-07-05

**Authors:** Nicholas E. Larkey, Radwa Ewaisha, Michael A. Lasho, Matthew M. Roforth, Dane Granger, Calvin R. Jerde, Liang Wu, Amy Gorsh, Stephen A. Klassen, Jonathon W. Senefeld, Michael J. Joyner, Nikola A. Baumann, Elitza S. Theel, John R. Mills

**Affiliations:** a Division of Laboratory Medicine and Pathology, Mayo Clinic, Rochester, Minnesota, USA; b Department of Microbiology and Immunology, Faculty of Pharmacy, Alexandria University, Alexandria, Egypt; c Advanced Diagnostics Laboratory, Mayo Clinic, Rochester, Minnesota, USA; d Department of Anesthesiology and Perioperative Medicine, Mayo Clinic, Rochester, Minnesota, USA; Montefiore Medical Center and Albert Einstein College of Medicine

**Keywords:** COVID-19 convalescent plasma, SARS-CoV-2, serology, antibody, CCP, FDA EUA, assay standardization, high-titer convalescent plasma, serological assays

## Abstract

In August 2020, the Food and Drug Administration (FDA) Emergency Use Authorization (EUA) for COVID-19 convalescent plasma (CCP) specified 12 authorized serologic assays and associated assay-specific cutoff values for the selection of high-titer CCP for use in hospitalized patients. The criteria used for establishing these cutoff values remains unclear. Here, we compare the overall agreement and concordance of five serologic assays included in the August 2020 FDA EUA at both the manufacturer-recommended qualitative cutoff thresholds and at the FDA-indicated thresholds for high-titer CCP, using serum samples collected as part of the CCP Expanded Access Program (EAP). The qualitative positive percent agreement (PPA) across assays ranged from 92.3% to 98.8%. However, the high-titer categorization across assays varied significantly, with the PPA ranging from 26.5% to 82.7%. The Roche anti-NC ECLIA provided the lowest agreement compared to all other assays. Efforts to optimize high-titer cutoffs could reduce, although not eliminate, the discordance across assays. The consequences of using nonstandardized assays are apparent in our study, and the high-titer cutoffs chosen for each assay are not directly comparable to each other. The generalized findings in our study will be relevant to any future use of convalescent plasma for either COVID-19 or future pandemics of newly emerged pathogens.

**IMPORTANCE** COVID-19 convalescent plasma (CCP) was one of the first therapeutic options available for the treatment of SARS-CoV-2 infections and continues to be used selectively for immunosuppressed patients. Given the emergence of novel SARS-CoV-2 variants which are resistant to treatment with available monoclonal antibody (MAb) therapy, CCP remains an important therapeutic consideration. The FDA has released several emergency use authorizations (EUA) that have specified which serological assays can be used for qualification of CCP, as well as assay-specific cutoffs that must be used to identify high-titer CCP. In this study, a cohort of donor CCP was assessed across multiple serological assays which received FDA EUA for qualification of CCP. This study indicates a high degree of discordance across the assays used to qualify CCP for clinical use, which may have precluded the optimal use of CCP, including during clinical trials. This study highlights the need for assay standardization early in the development of serological assays for emerging pathogens.

## INTRODUCTION

Early in the pandemic, the primary clinical use of SARS-CoV-2 antibody testing, as specified by the Centers for Disease Control and Prevention (CDC), was the identification and qualification of high-titer COVID-19 convalescent plasma (CCP) from recovered donors (1). CCP was the focus of a large-scale, national effort to collect convalescent plasma and was one of the first approaches taken to treat COVID-19, given its ability to be rapidly deployed ahead of vaccine development or monoclonal antibody (MAb) therapy (2). Individuals who recover from COVID-19 develop antibodies capable of neutralizing SARS-CoV-2, and the transfer of plasma containing these neutralizing antibodies to recipients early in the course of infection was postulated to potentially reduce disease mortality. However, robust methods for qualifying convalescent plasma units with maximum protective capacity has proven to be challenging. The influence of antibody specificities in suppressing SARS-CoV-2, the variability of antibody production and persistence, the differences associated with immune responses to SARS-CoV-2 variants, and the optimal methods for antibody measurement have yet to be fully understood and add to the complexity of CCP qualification (3–6).

Preliminary guidance from the FDA authorized the use of CCP with neutralizing antibody titers ≥ 1:160 for therapy in hospitalized COVID-19 patients early in their course of disease. At the time, there were no neutralizing antibody assays available that were capable of screening large numbers of samples, no established reference methodology against which to compare, and limited evidence that a 1:160 titer cutoff provided optimal efficacy. On August 23^rd^, 2020, following the analysis of data collected through the CCP Expanded Access Program (EAP), the FDA issued an EUA for the use of high-titer CCP defined as plasma units with a signal to cutoff (S/C) of ≥12 on the qualitative Ortho-Clinical anti-SARS-CoV-2 IgG assay. Subsequently, on February 4th, 2021, the FDA reissued their authorization but stipulated that only manufactured high-titer CCP, as defined on a per-assay basis using assay-specific cutoffs, was authorized for use in hospitalized patients (7). Since then, the FDA has released a series of EUA reauthorizations to revise the list of tests acceptable for use in the manufacture of high-titer CCP. Over this time, approved assays have included one surrogate neutralization assay (GenScript cPass SARS-CoV-2 Neutralization Antibody Kit), two immunoassays that detect antibodies against the SARS-CoV-2 nucleocapsid (NC) protein (Abbott SARS-CoV-2 IgG and the Roche Elecsys Anti-SARS-CoV-2), and numerous immunoassays that detect antibodies against all or part of the SARS-CoV-2 spike (S) glycoprotein. While these assays are more readily available compared to viral neutralizing antibody assays, to our knowledge, the parameters used to define these high-titer assay-specific cutoffs have not been reported in peer-reviewed literature.

Serological assays, including those that detect binding IgG antibodies targeting the RBD/S domains, have been reported to have various degrees of correlation with viral neutralization (6, 8–10). Given the lack of assay standardization, the heterogeneity of the assay formats, and the expedited process of assay development and validation under the EUA, there is concern that the degree of concordance between assays for the qualification of CCP as high-titer may be poor.

At this time, meta-analyses of clinical trials have mixed conclusions on the effectiveness of CCP against COVID-19 mortality (11–13). As of December 2021, the recommended use of CCP is restricted to COVID-19 patients with an immunosuppressive disease and those receiving immunosuppressive therapies (14). It remains unclear how the criteria used to define CCP as high-titer may have impacted the assessment of CCP effectiveness against COVID-19. The purpose of this study is to compare the overall agreement and concordance of five serologic assays with the FDA EUA for CCP qualification at both manufacturer-recommended qualitative cutoff thresholds and at the FDA-indicated thresholds for high-titer CCP.

## RESULTS

### CCP donor characteristics.

Serum was obtained from 1,005 CCP donors between 04/16/2020 and 8/19/2020. All samples were tested using the Roche anti-NC ECLIA and anti-S ECLIA and are defined as cohort 1. Donor age and sex were available on a subset of donors from cohort 1 (*n* = 594). The median age was 48 (range 16 to 81 years) and 47.8% (*n* = 284) were female. These 594 donor samples were also tested using the Ortho anti-S IgG CLIA. This subset of donors is defined as cohort 2. A subset of donors from cohort 2 (*n* = 187) were also tested on the Abbott anti-NC and Genscript cPass nAb assays. The median age and sex associated with these donors was 50 years (range 20 to 75 years) and 49.7% (*n* = 93) were female. These 187 donor samples were tested on all 5 assays included in this study and are defined as cohort 3.

### Qualitative positive agreement across SARS-CoV-2 serologic assays.

In cohort 1, 91.8% and 93.8% of the samples were positive for antibodies against SARS-CoV-2 by the Roche anti-NC and anti-S assays, respectively (Table S1). Using the anti-S assay as the reference assay, the positive percent agreement (PPA) between these two assays was 97.7% and was comparable to cohorts 2 and 3 (97.3% and 98.2%, respectively) (Table S2). In cohort 3, the Abbott anti-NC assay was associated with the lowest positivity rate (84%), whereas the Roche and Ortho anti-S assays had the highest positivity rates (both at 90.4%) (Table S1). The PPA across assays ranged from 92.3% (Genscript cPass nAb and Abbott anti-NC IgG CLIA) to 98.8% (Roche anti-S ECLIA and Ortho anti-S IgG CLIA) (Table S2).

### Agreement of SARS-CoV-2 serologic assays to categorize CCP as high-titer.

**FIG 1:**
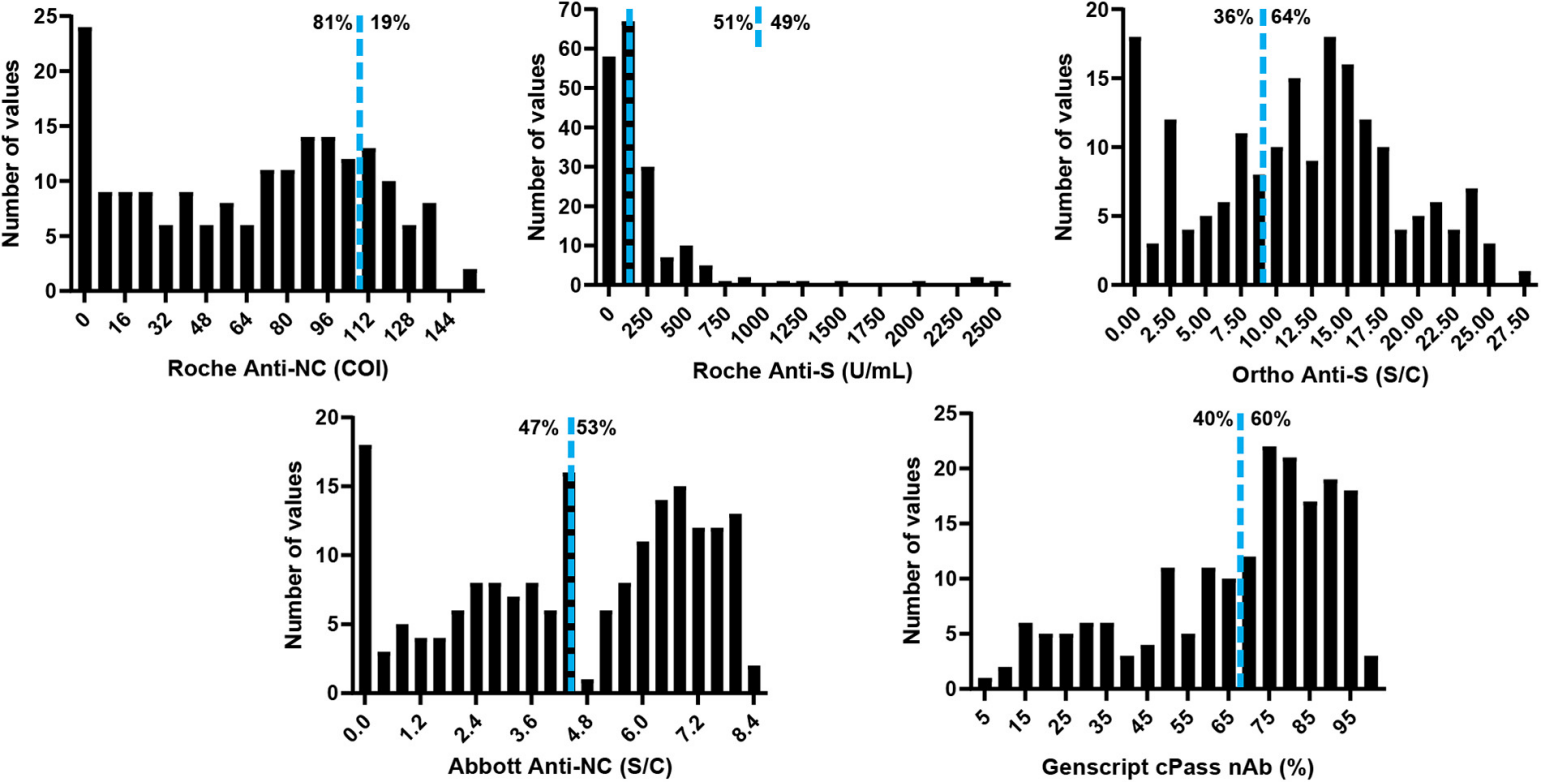
Histograms for each COVID-19 serology assay in cohort 3 (*n* = 187). The blue dashed line indicates the high-titer cutoff for each assay. Percentages to the left and right of the high-titer cutoff indicate the percentages of results that were below and above the high-titer cutoff, respectively.

**FIG 2:**
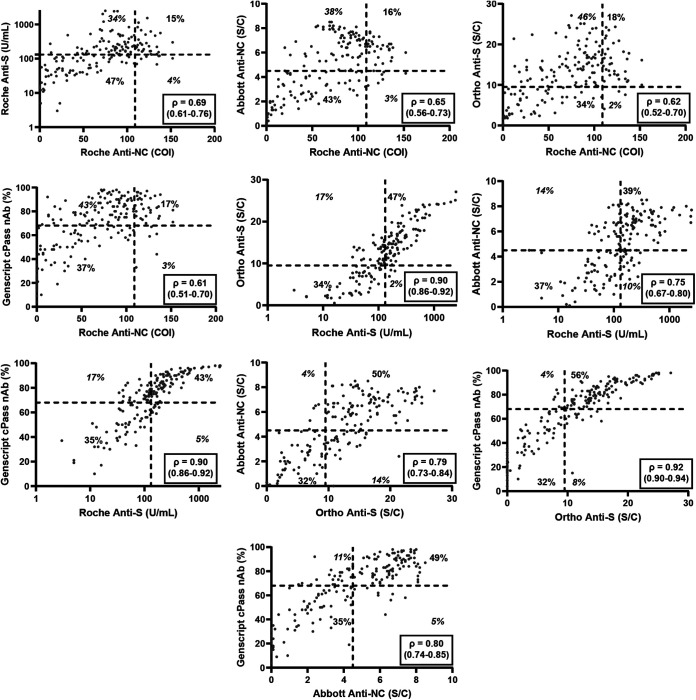
Correlation plots between COVID-19 serology assays in cohort 3 (*n* = 187). The dashed lines indicate the high-titer cutoffs for each assay, splitting the plots into four quadrants. Percentages indicate agreement and disagreement (italicized) percentages. The Spearman’s Rho correlation coefficient and 95% confidence interval for each pair are included. Roche anti-S was displayed in a logarithmic fashion and does not include any points <1 U/mL on the plot, but the included calculated values incorporate the missing points.

In contrast to the high PPA for the qualitative categorization of CCP as positive or negative using the manufacturer’s cutoff, the categorization of donor samples as high-titer per the FDA EUA assay-specific thresholds varied significantly across assays (Fig. 1 and Table S2). In cohort 1, the two Roche assays demonstrated significant differences in the likelihood of a donor being classified as high-titer. The NC-based assay identified only 19.0% of samples as high-titer, while the S-based assay indicated that 45.1% of donors were high-titer. Consistent with this finding, the high-titer agreement between these two assays was only 26.5%. In cohort 3, where samples were tested by five assays, the percentage of donor samples designated as high-titer ranged from 19.3% (Roche anti-NC ECLIA) to 64.2% (Ortho Anti-S IgG CLIA). The high-titer CCP percent agreement ranged from 26.5% (Roche anti-NC ECLIA and Genscript cPass nAb) to 82.7% (Ortho Anti-S IgG CLIA and Genscript cPass nAb). Correlation plots of the five assays utilized in cohort 3 show a wide range of agreement among assays (Fig. 2). Correlation, as determined by Spearman’s Rho, ranged from 0.61 when comparing the Roche anti-NC ECLIA and Genscript cPass nAb assays to 0.92 when comparing the Ortho anti-S IgG CLIA and Genscript cPass nAb assays.

### Optimized assay-specific high-titer cutoffs.

**FIG 3:**
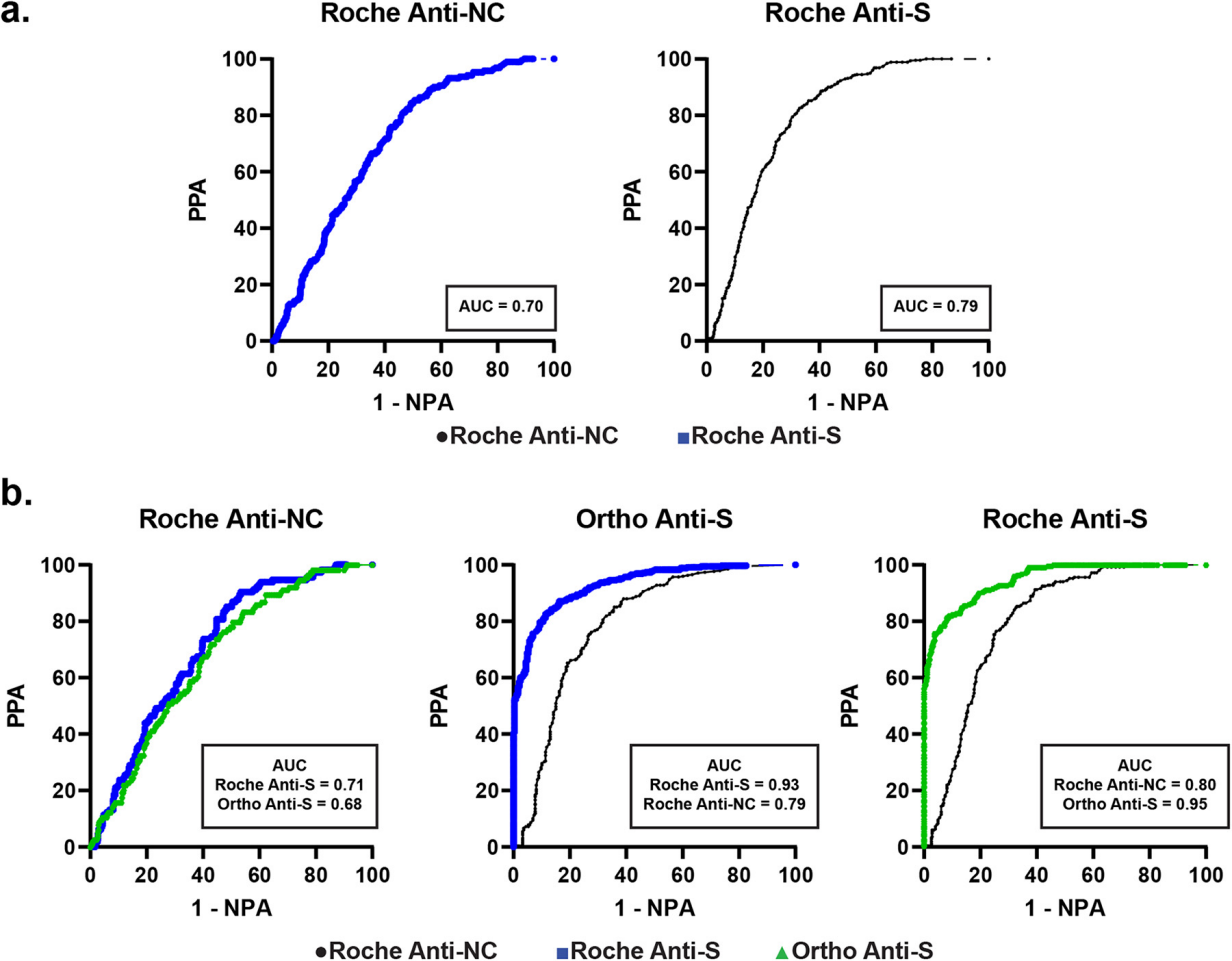
Receiver operating characteristic curves for cohorts 1 (a) and 2 (b). Percent positive and negative agreement were computed for each assay pair, using the indicated assay as the reference assay. The Roche anti-NC ECLIA assay is represented by black dots, the Roche anti-S ECLIA assay by blue squares, and the Ortho anti-S CLIA assay by green triangles. Areas under the curve (AUCs) are included in boxes.

**FIG 4:**
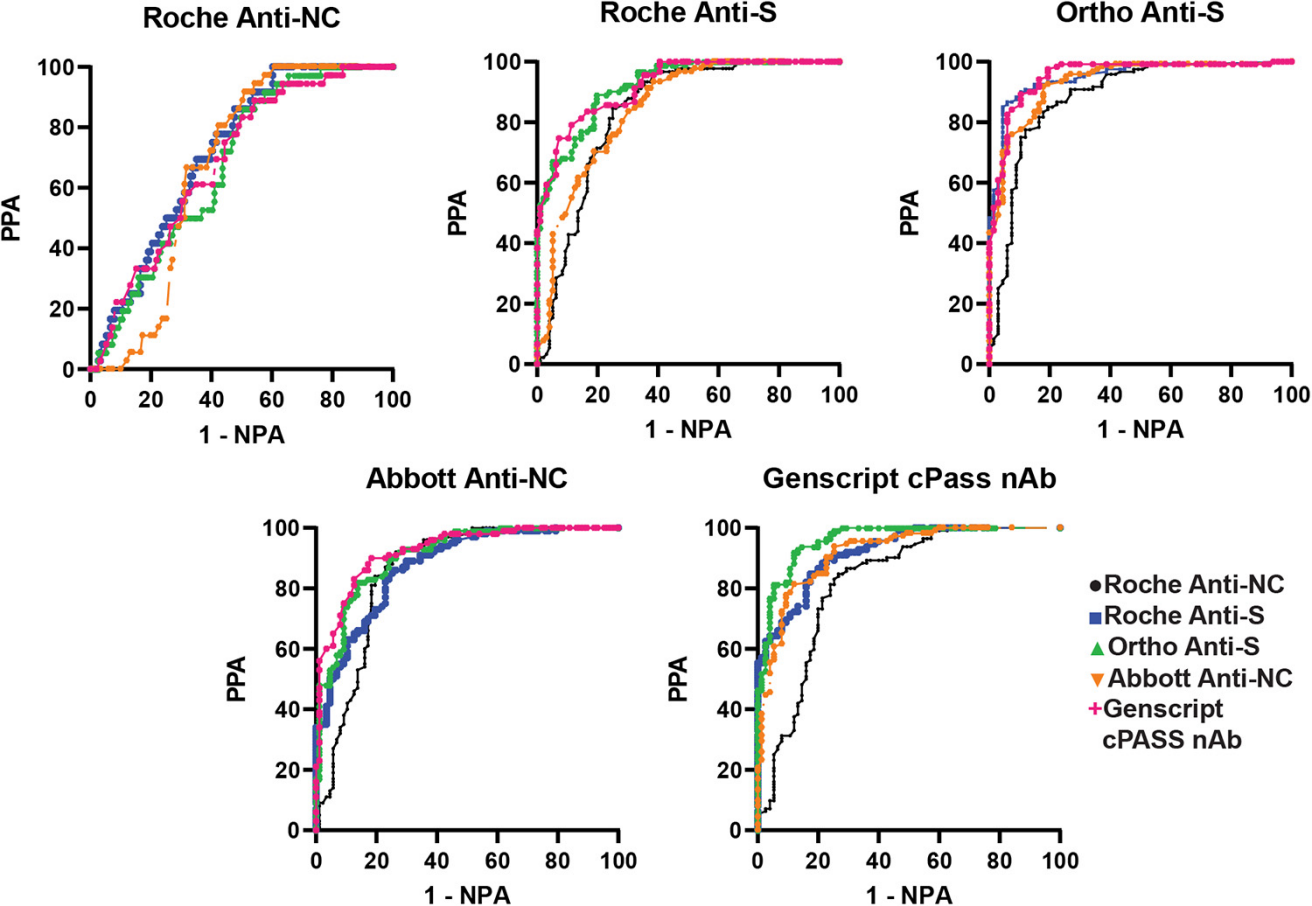
Receiver operating characteristic curves for cohort 3. Percent positive and negative agreement were computed for each assay pair, using the indicated assay as the reference assay. The Roche anti-NC ECLIA assay is represented by black dots, the Roche anti-S ECLIA assay by blue squares, the Ortho anti-S CLIA assay by green triangles, the Abbott anti-NC CLIA assay by orange inverted triangles, and the Genscript cPass nAb by pink plus signs. Areas under the curve (AUCs) can be found in Table S3.

In the absence of a reference method or the designation of a gold-standard method for the determination of high-titer CCP, we assessed agreement between each assay, across each cohort using receiver operator characteristic (ROC) curve analysis. Using each assay as the reference assay, PPA, NPA, and the area under the curve (AUC) were calculated for the comparator assay in each pair. ROC curves were generated for each method using different reference assays in each of the three sample cohorts (Fig. 3 and 4). In cohort 1, the AUC was 0.70 when using the Roche anti-S ECLIA assay as the comparator and the Roche anti-NC ECLIA assay as the reference assay (Fig. 3a and Table S3). The optimized high-titer cutoff was determined to be 115 U/mL using the equation below (see Materials and Methods) (88 to 159 U/mL using the two alternate methods) (Table S3), which is slightly lower than the EUA cutoff of 132 U/mL. Conversely, the Roche anti-NC ECLIA had an AUC of 0.79 when using the Roche anti-S ECLIA as the reference assay. The ideal high-titer cutoff for Roche anti-NC was determined to be a cutoff index (COI) of 58 (53 to 65 using the two alternative methods) (Table S3), which is notably lower than the FDA EUA cutoff of 109 for high-titer CCP.

In cohort 2 (Fig. 3b), the Roche anti-NC ECLIA, Roche anti-S ECLIA, and Ortho anti-S CLIA assays were each compared. The Roche anti-S ECLIA and Ortho anti-S CLIA assays correlated better to each other than to the Roche anti-NC at their FDA EUA established high-titer cutoffs. The Roche anti-S assay ECLIA and Ortho anti-S CLIA AUCs were 0.93 to 0.95 compared with each other, and 0.71 to 0.80 and 0.68 to 0.79, respectively, compared with the Roche anti-NC method (Table S3).

In cohort 3 (Fig. 4), all five assays were compared. Similar to the findings above, the Roche anti-NC ECLIA had the lowest AUCs (0.67 to 0.72) when used as the reference assay. When the other assays were used as the reference assay, the same trend was observed, with the Roche anti-NC ECLIA generating the lowest AUCs (0.82 to 0.88). In comparison, AUCs among each of the other four assays ranged from 0.85 to 0.96 (Table S3). Depending on the comparator assay and the reference assay, we found that the calculated cutoffs could vary considerably from the FDA EUA high-titer cutoff (Table S4).

### Statistical analysis by age and sex.

Additional statistical comparisons were performed (Tables S5–S8). There were no significant differences in the antibody titers per assay based on sex, although the males tended to be slightly older than the females in each cohort (Tables S6 and S8). Older age was associated with higher antibody titers in all cohorts, although this trend did not reach statistical significance for all assays. Consistent with this finding, high-titer CCP, according to the FDA EUA cutoffs, was more frequently observed among older donors (>55 years) for all assays in all cohorts compared to younger donors (<40 years). However, the trend did not achieve statistical significance for all assays (Tables S5 and S7).

## DISCUSSION

Early in the SARS-CoV-2 pandemic, CCP was a frequently relied on therapy, particularly prior to the development of monoclonal antibody therapy and the deployment of vaccines. The identification of potential CCP donors and the qualification of CCP evolved during the pandemic as more information became available about both COVID-19 and the performance of serological assays and as the availability of neutralizing antibody assays increased. This was reflected in multiple FDA EUA updates related to the use of CCP, in which the laboratory testing requirements for CCP qualification were modified.

Several studies have compared the performance of different serologic assays for the purposes of categorizing CCP as high-titer (15–18). Our study builds on prior reports by showing that although the five serologic assays evaluated here had comparable qualitative performance for the identification of antibody-positive CCP samples, limited correlation was observed for the classification of CCP as high-titer using the FDA EUA specific cutoff values. Most notably, the Roche anti-NC ECLIA assay showed the lowest correlation with high-titer CCP classification, both relative to the FDA EUA threshold and compared to other binding antibody assays. This assay had the lowest AUC across all comparisons, suggesting that this assay can identify a donor population that is distinct from that identified by the other assays. This raises the question of whether utilizing NC-based serologic assays for the identification of high-titer CCP is appropriate, as the therapeutic benefit of CCP is considered to manifest through the disruption of binding interactions between the SARS-CoV-2 spike protein and the host cell ACE2 receptor. Using our study cohort to optimize high-titer cutoffs to reduce discordance across assays, we were only able to partially reduce the disagreement across assays. We noticed relatively large differences between the FDA EUA recommended cutoffs and our optimized cutoffs, which suggests that the methods used to identify the high-titer threshold are likely different between our study and that of the FDA. The majority of commercially available serological assays available within the first 2 years of the pandemic were designed and optimized to qualitatively detect binding antibodies, not as correlates of antibody neutralization. Thus, manufacturer cutoffs were of negligible utility for the detection of neutralizing antibody activity. Initial studies performed by Biomedical Advanced Research and Development Authority (BARDA), using the EAP data, demonstrated that qualitative positivity on serological binding assays alone was not a robust predictor of neutralizing antibody titers. Thus, it was recommended that alternative cutoffs be utilized for the qualification of high-titers. Subsequent studies have demonstrated variable assay performance when attempting to correlate qualitative assay cutoffs to various neutralizing activities measured using neutralization assays (19).

The consequences of using nonstandardized assays are apparent in our study, and it is clear that the high-titer cutoffs chosen for each assay are not directly comparable to each other. This is an issue that could have been mitigated by better standardization across assays when determining these high-titer cutoffs. Interestingly, on December 28^th^ of 2021, a new FDA EUA relating to the use of CCP was issued, and the list of authorized assays for qualifying high-titer CCP was modified to exclude the use of the qualitative assays, including the Roche anti-NC ECLIA, the Ortho anti-S CLIA, and the Abbott anti-NC CLIA assays. The Roche anti-S ECLIA and GenScript cPass neutralizing antibody assays had their high-titer cutoffs increased from 132 U/mL to 210 U/mL and 68% to 80%, respectively. The impact of these changes reduced the number of CCP units with the high-titer designation and improved the qualitative agreement between these two assays (19).

The World Health Organization has developed international standards for anti-SARS-CoV-2 antibodies (20). These standards can be used for the calibration of quantitative tests and the correlation of numeric antibody results. The use of calibrated control material in all assays is the first step to achieve standardization. While standardization will help ensure comparability across platforms, the rapid emergence of SARS-CoV-2 variants has the potential to undermine these efforts. Mutations in the spike protein enables antigenic escape and raises the concern of whether the SARS-CoV-2 antigens utilized in currently-deployed serological assays will be sufficient to gauge the effectiveness of CCP. Therefore, in addition to standardization efforts, there is also a need to rapidly assess what impact new variants may have on serological assays (21). The generalized findings in our study will be relevant to the future qualification of convalescent plasma for either COVID-19 or future pandemics of newly emerged pathogens.

## MATERIALS AND METHODS

### Study design and patient samples.

Residual waste serum samples (*n* = 1,005) tested as part of the COVID-19 Convalescent Plasma Expanded Access Program were utilized. Inclusion criteria were defined by the CCP EAP study. Donors were required to have a prior infection confirmed by SARS-CoV-2 RT-PCR, and samples had to be collected at least 14 days after symptom resolution (22). Samples were collected, following standardized blood-collection procedures, at all centers (23). Following collection, the samples were aliquoted and immediately stored at −80°C for up to 6 months prior to testing. The study was approved by the Mayo Clinic Institutional Review Board.

### SARS-CoV-2 serological assays.

Antibody titers in serum specimens were measured using up to five assays, all of which received FDA EUA for use in the manufacture of high-titer CCP. The Roche Elecsys anti-SARS-CoV-2 assay (Indianapolis, IN) is an electrochemiluminescent immunoassay (ECLIA) that qualitatively detects total antibodies to the nucleocapsid (NC) protein and was performed on a Cobas e801. The Roche Elecsys anti-SARS-CoV-2 S assay is a semiquantitative ECLIA that detects antibodies to the receptor-binding domain (RBD) of the S glycoprotein and was also performed using the Cobas e801. The Ortho-Clinical anti-SARS-CoV-2 IgG assay (Rochester, NY) is a chemiluminescent immunoassay (CLIA) that qualitatively detects IgG against subunit 1 of the S glycoprotein and was performed on the VITROS 3600 instrument. The Genscript cPass SARS-CoV-2 Neutralization Antibody (nAb) Detection Kit measures the capacity of patient antibodies to inhibit the RBD:ACE2 interaction *in vitro* and was performed manually. The Abbott SARS-CoV-2 IgG CLIA (Abbott Park, IL) qualitatively detects IgG to the NC protein and was performed on an Abbott Architect i2000. All tests were performed without deviation from manufacturer instructions. Of the 1,005 samples included in the study, 411 were only tested by the two Roche assays, 407 were tested on both Roche assays and the Ortho-Clinical assay only, and 187 were tested on all five assays. This gave us the following three cohorts: cohort 1 (*n* = 1,005) for Roche anti-NC ECLIA and Roche anti-S ECLIA, cohort 2 (*n* = 594) for Ortho anti-S IgG CLIA, Roche anti-NC ECLIA and Roche anti-S ECLIA, and cohort 3 (*n* = 187) for Ortho anti-S IgG CLIA, Roche anti-NC ECLIA, and Roche anti-S ECLIA, Genscript cPass nAb, and Abbott IgG anti-NC CLIA. The manufacturer determined positive cutoffs for the Ortho anti-S IgG CLIA, Roche anti-NC ECLIA, Roche anti-S ECLIA, Genscript cPass nAb, and Abbott IgG anti-NC CLIA are ≥1.00 signal to cutoff (S/C), ≥1.0 cutoff index (COI), ≥0.8 U/mL, ≥30% signal inhibition (SI), and ≥ 1.40 S/C, respectively, whereas the FDA endorsed high-titer CCP thresholds are ≥9.5 S/C, ≥109 COI, ≥132 U/mL, ≥68% SI, and ≥ 4.5 (S/C), respectively (Table 1).

**TABLE 1:** Positivity and EUA high-titer cutoffs for the SARS-CoV-2 serologic assays used

Manufacturer	Assay	Assay description	Positivity/reactivity cutoff^*a*^	EUA High-Titer cutoff^*b*^
Abbott	SARS-CoV-2 IgG	Anti-nucleocapsid	Index (S/C) ≥ 1.40	Index (S/C) ≥ 4.5
GenScript	cPass SARS-CoV-2 Neutralization Antibody	Neutralizing antibodies	Inhibition ≥ 30%	Inhibition ≥ 68%
Ortho	VITROS Anti-SARS-CoV-2 IgG	Antibodies against subunit 1 of the spike protein	S/C ≥ 1.00	S/C ≥ 9.5
Roche	Elecsys Anti-SARS-CoV-2	Anti-nucleocapsid	Cutoff index > 1.0	Cutoff index ≥ 109
Roche	Elecsys Anti-SARS-CoV-2 S	Anti-spike	≥0.80 U/mL	≥132 U/mL

a According to package insert.

b According to FDA EUA for CCP issued August 2020.

### Statistical analysis.

For each test, both the numerical results and the qualitative interpretation for high-titer/low-titer were compared. Numerical results from a subset of donors were compared by categorized ages (<40 years, 40 to <55 years, and >55 years) and sex (M/F). For numerical results, the median, first quartile (Q1), and third quartile (Q3) were determined for each assay. *P*-values were determined for each assay using either chi-square or Kruskal-Wallis tests. ROC curves with their associated AUC analyses were generated using GraphPad Prism 9 (San Diego, CA), using the FDA EUA high-titer cutoffs for CCP. In the ROC curves, positive percent agreement (PPA) is plotted against one minus the negative percent agreement (NPA). The PPA and NPA terms are used instead of sensitivity and specificity due to the error associated with each assay. The ROC curves were used to establish optimized cutoff values via the equation below, for which the minimum value determines which cutoff point minimizes the distance from the ROC curve to the point (0, 1) on the chart (24).

$$\sqrt{{\left(1-\mathrm{PPA}\right)}^{2}+{\left(1-\mathrm{NPA}\right)}^{2}}$$

Further evaluation of potential high-titer cutoffs by minimizing the absolute value of PPA-NPA or maximizing Youden’s index (J) were also performed (Table S3) (25).
