# Supplementary material for: Limited Correlation between SARS-CoV-2 Serologic Assays for Identification of High-Titer COVID-19 Convalescent Plasma Using FDA Thresholds
Source: Microbiol Spectr. 2022 Jul 5;10(4):e01154-22. doi: 10.1128/spectrum.01154-22 (PMC9430146; doi:10.1128/spectrum.01154-22)
Supplement: Supplemental file 1 — Tables S1 to S8. Download spectrum.01154-22-s0001.pdf, PDF file, 0.7 MB [file spectrum.01154-22-s0001.pdf]

Supplemental Table 1. Total number and sample percent positivity for each cohort.

**Samples**	**Assay**	**Positive (%)**	**High-Titer (%)**
**Cohort 1** (N=1005)	Roche Anti-NC	923 (91.8)	191 (19.0)
	Roche Anti-S	943 (93.8)	453 (45.1)
**Cohort 2** N=594	Roche Anti-NC	536 (90.2)	114 (19.2)
	Roche Anti-S	549 (92.4)	251 (42.3)
	Ortho Anti-S	555 (93.4)	348 (58.6)
**Cohort 3** N=187	Roche Anti-NC	166 (88.8)	36 (19.3)
	Roche Anti-S	169 (90.4)	91 (48.7)
	Ortho Anti-S	169 (90.4)	120 (64.2)
	Abbott Anti-NC	157 (84.0)	100 (53.5)
	Genscript cPass nAb	168 (89.8)	112 (59.9)

Supplemental Table 2. Positive percent agreement and high-titer percent agreement between assays for each cohort.

**Cohort 1**				
**Positive % Agreement**	Roche Anti-S			
Roche Anti-NC	97.7%			
**High-Titer % Agreement**	Roche Anti-S			
Roche Anti-NC	26.5%			
**Cohort 2**				
**Positive % Agreement**	Roche Anti-S	Ortho Anti-S		
Roche Anti-NC	97.3%	95.2%		
Roche Anti-S		97.1%		
**High-Titer % Agreement**	Roche Anti-S	Ortho Anti-S		
Roche Anti-NC	26.3%	24.9%		
Roche Anti-S		66.4%		
**Cohort 3**				
**Positive % Agreement**	Roche Anti-S	Ortho Anti-S	Abbott Anti-NC	Genscript cPass nAb
Roche Anti-NC	98.2%	98.2%	94.6%	96.5%
Roche Anti-S		98.8%	92.9%	95.9%
Ortho Anti-S			92.9%	95.9%
Abbott Anti-NC				92.3%
**High-Titer % Agreement**	Roche Anti-S	Ortho Anti-S	Abbott Anti-NC	Genscript cPass nAb

Roche Anti-NC	28.3%	26.8%	28.3%	26.5%
Roche Anti-S		71.5%	61.9%	66.4%
Ortho Anti-S			73.2%	82.7%
Abbott Anti-NC				75.2%

Supplemental Table 3. ROC data and new calculated high-titer cutoffs

N	Gold Standard	Test	AUC	Std. Error	95% CI	P value	Max (PPA+NPA-1) Youden's index	Min |PPA-NPA|	Min $\sqrt{(1-PPA)^2+(1-NPA)^2}$
**1005 (Cohort 1)**	**Roche Anti-NC**	**Roche Anti-S**	0.7034	0.01842	0.6673 to 0.7396	<0.001	88	159	115
	**Roche Anti-S**	**Roche Anti-NC**	0.7937	0.01414	0.7660 to 0.8215	<0.0001	53	65	58
**594 (Cohort 2)**	**Roche Anti-NC**	**Roche Anti-S**	0.7105	0.02374	0.6639 to 0.7570	<0.001	68	136	114
		**Ortho Anti-S**	0.6758	0.02521	0.6264 to 0.7252	<0.001	10	12.5	11.4
	**Roche Anti-S**	**Roche Anti-NC**	0.8018	0.01806	0.7664 to 0.8372	<0.001	52	69	66
		**Ortho Anti-S**	0.9478	0.007926	0.9322 to 0.9633	<0.001	13.6	12.2	13.3
	**Ortho Anti-S**	**Roche Anti-NC**	0.7912	0.02019	0.7517 to 0.8308	<0.001	32	54	51
		**Roche Anti-S**	0.9299	0.009811	0.9107 to 0.9491	<0.001	92	84	88
**187 (Cohort 3)**	**Roche Anti-NC**	**Roche Anti-S**	0.7182	0.03926	0.6412 to 0.7951	<0.001	68	161	155
		**Ortho Anti-S**	0.6756	0.04215	0.5930 to 0.7582	0.001	10.8	13.4	12.4
		**Abbott Anti-NC**	0.6718	0.03778	0.5978 to 0.7459	0.001	4.5	5.9	6
		**Genscript cPass nAb**	0.6878	0.04346	0.6026 to 0.7730	<0.001	67	77	74
	**Roche Anti-S**	**Roche Anti-NC**	0.8374	0.03038	0.7778 to 0.8969	<0.001	69	75	69

		**Ortho Anti-S**	0.9211	0.01812	0.8856 to 0.9566	<0.001	11.3	12.4	11.3
		**Abbott Anti-NC**	0.8471	0.0282	0.7919 to 0.9024	<0.001	3.5	5	4.5
		**Genscript cPass nAb**	0.922	0.01806	0.8866 to 0.9574	<0.001	77	75	75
	**Ortho Anti-S**	**Roche Anti-NC**	0.8841	0.02841	0.8284 to 0.9398	<0.001	53	57	56
		**Roche Anti-S**	0.9491	0.01567	0.9184 to 0.9798	<0.001	108	99	97
		**Abbott Anti-NC**	0.9333	0.01842	0.8972 to 0.9694	<0.001	3.5	4.1	3.5
		**Genscript cPass nAb**	0.9527	0.01602	0.9213 to 0.9841	<0.001	67	67	67
	**Abbott Anti-NC**	**Roche Anti-NC**	0.8562	0.03005	0.7973 to 0.9151	<0.001	53	70	69
		**Roche Anti-S**	0.8753	0.02459	0.8272 to 0.9235	<0.001	108	126	115
		**Ortho Anti-S**	0.9094	0.02083	0.8685 to 0.9502	<0.001	12.2	11.5	12.2
		**Genscript cPass nAb**	0.9243	0.01874	0.8876 to 0.9610	<0.001	70	73	70
	**Genscript cPass nAb**	**Roche Anti-NC**	0.8208	0.03403	0.7541 to 0.8875	<0.001	53	65	58
		**Roche Anti-S**	0.9207	0.01846	0.8845 to 0.9568	<0.001	108	112	108
		**Ortho Anti-S**	0.9594	0.01325	0.9334 to 0.9854	<0.001	10.5	10.8	10.5
		**Abbott Anti-NC**	0.9164	0.02074	0.8758 to 0.9571	<0.001	4.9	4.4	4.5

Supplemental Table 4. Calculated difference between FDA EUA high-titer cutoff value and differences between calculated values from Supplemental Table 3.

N	Gold Standard	Test	EUA Value	Minimum Difference	Maximum Difference	Minimum Absolute Difference (%)
**1005 (Cohort 1)**	**Roche Anti-NC**	**Roche Anti-S**	132	-27	44	17 (13)
	**Roche Anti-S**	**Roche Anti-NC**	109	44	56	44 (40)
**594 (Cohort 2)**	**Roche Anti-NC**	**Roche Anti-S**	132	-4	64	4 (3)
		**Ortho Anti-S**	9.5	-3	-0.5	0.5 (5)
	**Roche Anti-S**	**Roche Anti-NC**	109	40	57	40 (37)
		**Ortho Anti-S**	9.5	-4.1	-2.7	2.7 (28)
	**Ortho Anti-S**	**Roche Anti-NC**	109	55	77	55 (50)
		**Roche Anti-S**	132	40	48	40 (30)
**187 (Cohort 3)**	**Roche Anti-NC**	**Roche Anti-S**	132	-29	64	23 (17)
		**Ortho Anti-S**	9.5	-3.9	-1.3	1.3 (14)
		**Abbott Anti-NC**	4.5	-1.5	0	0 (0)
		**Genscript cPass nAb**	68	-9	1	1 (1)
	**Roche Anti-S**	**Roche Anti-NC**	109	34	40	34 (31)
		**Ortho Anti-S**	9.5	-2.9	-1.8	1.8 (19)
		**Abbott Anti-NC**	4.5	-0.5	1	0 (0)
		**Genscript cPass nAb**	68	-9	-7	7 (10)
	**Ortho Anti-S**	**Roche Anti-NC**	109	52	56	52 (48)
		**Roche Anti-S**	132	24	35	24 (18)
		**Abbott Anti-NC**	4.5	0.4	1	0.4 (9)
		**Genscript cPass nAb**	68	1	1	1 (1)
	**Abbott Anti-NC**	**Roche Anti-NC**	109	39	56	39 (36)
		**Roche Anti-S**	132	6	24	6 (5)
		**Ortho Anti-S**	9.5	-2.7	-2	2 (21)

		**Genscript cPass nAb**	68	-5	-2	2 (3)
	**Genscript cPass nAb**	**Roche Anti-NC**	109	44	56	44 (40)
		**Roche Anti-S**	132	20	24	20 (15)
		**Ortho Anti-S**	9.5	-1.3	-1	1 (11)
		**Abbott Anti-NC**	4.5	-0.4	0.1	0 (0)

Supplemental Table 5. Cohort 2 statistics by age category

Summary of 594 samples tested across three assays
By age category

	1) <40 (N=237)	2) 40 to <55 (N=147)	3) >=55 (N=210)	Total (N=594)	p value
**Sex**					0.0169[1]
F	129 (54.4%)	69 (46.9%)	86 (41.0%)	284 (47.8%)	
M	108 (45.6%)	78 (53.1%)	124 (59.0%)	310 (52.2%)	
**Ortho [VITROS Anti-SARS-CoV-2 IgG]**					0.0001[2]
Median	9.9	12.4	12.5	11.1	
Q1, Q3	4.8, 14.0	4.8, 18.2	6.0, 18.7	5.3, 17.0	
Range	(0.0-28.9)	(0.0-28.4)	(0.0-29.5)	(0.0-29.5)	
**Ortho [VITROS Anti-SARS-CoV-2 IgG]: High titer**					0.0875[1]
0) S/C<9.5	111 (46.8%)	57 (38.8%)	78 (37.1%)	246 (41.4%)	
1) S/C>=9.5	126 (53.2%)	90 (61.2%)	132 (62.9%)	348 (58.6%)	
**Roche COI [Elecsys Anti-SARS-CoV-2]**					0.0532[2]
Median	52.9	65.4	71.0	61.1	
Q1, Q3	9.8, 97.0	16.9, 99.7	23.1, 103.0	16.9, 99.9	
Range	(0.1-160.0)	(0.1-167.0)	(0.1-152.0)	(0.1-167.0)	
**Roche COI [Elecsys Anti-SARS-CoV-2]: High titer**					0.3914[1]
0) COI<109	191 (80.6%)	124 (84.4%)	165 (78.6%)	480 (80.8%)	
1) COI>=109	46 (19.4%)	23 (15.6%)	45 (21.4%)	114 (19.2%)	
**Roche U/mL [Elecsys Anti-SARS-CoV-2 S]**					0.0003[2]
Median	84.6	104.0	143.5	102.5	
Q1, Q3	22.7, 168.0	30.6, 277.6	40.0, 295.2	32.4, 238.4	
Range	(0.4-2230.0)	(0.4-2501.0)	(0.4-2501.0)	(0.4-2501.0)	
**Roche U/mL [Elecsys Anti-SARS-CoV-2 S]: High titer**					0.0008[1]
0) <132 U/mL	157 (66.2%)	84 (57.1%)	102 (48.6%)	343 (57.7%)	
1) >=132 U/mL	80 (33.8%)	63 (42.9%)	108 (51.4%)	251 (42.3%)	

(report generated on 26APR2021)
[1]Chi-Square [2]Kruskal Wallis

Supplemental Table 6. Cohort 2 statistics by sex

Summary of 594 samples tested across three assays
By sex

	F (N=284)	M (N=310)	Total (N=594)	p value
**Age**				0.0059[1]
Median	42.5	49.2	47.8	
Q1, Q3	28.2, 57.8	32.4, 59.4	31.0, 59.0	
Range	(16.3-73.1)	(17.2-81.2)	(16.3-81.2)	
**Age (category)**				0.0169[2]
1) <40	129 (45.4%)	108 (34.8%)	237 (39.9%)	
2) 40 to <55	69 (24.3%)	78 (25.2%)	147 (24.7%)	
3) >=55	86 (30.3%)	124 (40.0%)	210 (35.4%)	
**Ortho [VITROS Anti-SARS-CoV-2 IgG]**				0.5599[1]
Median	11.1	11.1	11.1	
Q1, Q3	5.3, 15.8	5.3, 17.7	5.3, 17.0	
Range	(0.0-29.5)	(0.0-28.2)	(0.0-29.5)	
**Ortho [VITROS Anti-SARS-CoV-2 IgG]: High titer**				0.5465[2]
0) S/C<9.5	114 (40.1%)	132 (42.6%)	246 (41.4%)	
1) S/C>=9.5	170 (59.9%)	178 (57.4%)	348 (58.6%)	
**Roche COI [Elecsys Anti-SARS-CoV-2]**				0.9477[1]
Median	61.7	60.3	61.1	
Q1, Q3	16.2, 101.0	17.1, 99.3	16.9, 99.9	
Range	(0.1-160.0)	(0.1-167.0)	(0.1-167.0)	
**Roche COI [Elecsys Anti-SARS-CoV-2]: High titer**				0.4660[2]
0) COI<109	226 (79.6%)	254 (81.9%)	480 (80.8%)	
1) COI>=109	58 (20.4%)	56 (18.1%)	114 (19.2%)	
**Roche U/mL [Elecsys Anti-SARS-CoV-2 S]**				0.3605[1]
Median	90.8	108.5	102.5	
Q1, Q3	28.5, 224.8	34.9, 256.8	32.4, 238.4	
Range	(0.4-2501.0)	(0.4-2501.0)	(0.4-2501.0)	
**Roche U/mL [Elecsys Anti-SARS-CoV-2 S]: High titer**				0.4051[2]
0) <132 U/mL	169 (59.5%)	174 (56.1%)	343 (57.7%)	
1) >=132 U/mL	115 (40.5%)	136 (43.9%)	251 (42.3%)	

(report generated on 26APR2021)
[1]Wilcoxon [2]Chi-Square

Supplemental Table 7. Cohort 3 statistics by age category

Summary of 187 samples tested across six assays
By age category

	1) <40 (N=69)	2) 40 to <55 (N=48)	3) >=55 (N=70)	Total (N=187)	p value
**Sex**					0.0474[1]
F	37 (53.6%)	29 (60.4%)	27 (38.6%)	93 (49.7%)	
M	32 (46.4%)	19 (39.6%)	43 (61.4%)	94 (50.3%)	
**Ortho [VITROS Anti-SARS-CoV-2 IgG]**					0.0190[2]
Median	10.0	13.7	12.4	12.2	
Q1, Q3	3.0, 14.4	4.8, 17.7	7.8, 17.0	6.6, 16.5	
Range	(0.0-22.4)	(0.0-27.1)	(0.0-25.1)	(0.0-27.1)	
**Ortho [VITROS Anti-SARS-CoV-2 IgG]: High titer**					0.3920[1]
0) S/C<9.5	29 (42.0%)	16 (33.3%)	22 (31.4%)	67 (35.8%)	
1) S/C>=9.5	40 (58.0%)	32 (66.7%)	48 (68.6%)	120 (64.2%)	
**Roche COI [Elecsys Anti-SARS-CoV-2]**					0.0208[2]
Median	55.8	70.9	87.4	72.6	
Q1, Q3	13.4, 92.2	19.0, 99.8	43.1, 111.0	25.5, 101.0	
Range	(0.1-152.0)	(0.1-138.0)	(0.1-152.0)	(0.1-152.0)	
**Roche COI [Elecsys Anti-SARS-CoV-2]: High titer**					0.1789[1]
0) COI<109	57 (82.6%)	42 (87.5%)	52 (74.3%)	151 (80.7%)	
1) COI>=109	12 (17.4%)	6 (12.5%)	18 (25.7%)	36 (19.3%)	
**Roche U/mL [Elecsys Anti-SARS-CoV-2 S]**					0.0353[2]
Median	108.0	126.0	166.5	127.0	
Q1, Q3	38.7, 157.0	32.5, 297.2	60.0, 231.0	45.1, 229.0	
Range	(0.4-683.2)	(0.4-2501.0)	(0.4-2430.0)	(0.4-2501.0)	
**Roche U/mL [Elecsys Anti-SARS-CoV-2 S]: High titer**					0.0436[1]
0) <132 U/mL	42 (60.9%)	26 (54.2%)	28 (40.0%)	96 (51.3%)	
1) >=132 U/mL	27 (39.1%)	22 (45.8%)	42 (60.0%)	91 (48.7%)	
**Abbott [SARS-CoV-2 IgG]**					0.1339[2]
Median	4.3	5.0	5.5	4.9	
Q1, Q3	1.7, 6.5	2.1, 6.7	3.2, 7.0	2.5, 6.7	
Range	(0.0-8.5)	(0.0-8.2)	(0.0-7.9)	(0.0-8.5)	
**Abbott [SARS-CoV-2 IgG]: High titer**					0.4500[1]
0) Index (S/C)<4.5	37 (53.6%)	21 (43.8%)	31 (44.3%)	89 (47.6%)	
1) Index (S/C)>=4.5	32 (46.4%)	27 (56.3%)	39 (55.7%)	98 (52.4%)	
**GenScript cPass SARS-CoV-2 Neutralization Antibody]**					0.0100[2]

Summary of 187 samples tested across six assays
By age category

	1) <40 (N=69)	2) 40 to <55 (N=48)	3) >=55 (N=70)	Total (N=187)	p value
Median	66.8	77.4	78.4	74.0	
Q1, Q3	43.6, 79.1	47.5, 87.8	60.9, 89.2	51.0, 85.3	
Range	(12.9-96.3)	(4.2-98.0)	(9.4-97.6)	(4.2-98.0)	
**GenScript [cPass SARS-CoV-2 Neutralization Antibody]: High titer**					0.0575[1]
0) Inhibition<68%	36 (52.2%)	18 (37.5%)	23 (32.9%)	77 (41.2%)	
1) Inhibition>=68%	33 (47.8%)	30 (62.5%)	47 (67.1%)	110 (58.8%)	

(report generated on 26APR2021)
[1]Chi-Square [2]Kruskal Wallis

Supplemental Table 8. Cohort 3 statistics by sex

Summary of 187 samples tested across six assays
By sex

	F (N=93)	M (N=94)	Total (N=187)	p value
**Age**				0.1068[1]
Median	48.0	53.0	50.0	
Q1, Q3	33.0, 56.0	34.0, 60.0	34.0, 59.0	
Range	(20.0-70.0)	(21.0-75.0)	(20.0-75.0)	
**Age (category)**				0.0474[2]
1) <40	37 (39.8%)	32 (34.0%)	69 (36.9%)	
2) 40 to <55	29 (31.2%)	19 (20.2%)	48 (25.7%)	
3) >=55	27 (29.0%)	43 (45.7%)	70 (37.4%)	
**Ortho [VITROS Anti-SARS-CoV-2 IgG]**				0.8057[1]
Median	11.9	12.3	12.2	
Q1, Q3	6.9, 15.1	6.2, 16.7	6.6, 16.5	
Range	(0.0-27.1)	(0.0-25.1)	(0.0-27.1)	
**Ortho [VITROS Anti-SARS-CoV-2 IgG]: High titer**				0.8359[2]
0) S/C<9.5	34 (36.6%)	33 (35.1%)	67 (35.8%)	
1) S/C>=9.5	59 (63.4%)	61 (64.9%)	120 (64.2%)	
**Roche COI [Elecsys Anti-SARS-CoV-2]**				0.4114[1]
Median	71.1	79.2	72.6	
Q1, Q3	20.0, 99.9	35.0, 102.0	25.5, 101.0	
Range	(0.1-137.0)	(0.1-152.0)	(0.1-152.0)	
**Roche COI [Elecsys Anti-SARS-CoV-2]: High titer**				0.7374[2]
0) COI<109	76 (81.7%)	75 (79.8%)	151 (80.7%)	
1) COI>=109	17 (18.3%)	19 (20.2%)	36 (19.3%)	
**Roche U/mL [Elecsys Anti-SARS-CoV-2 S]**				0.8829[1]
Median	126.0	130.5	127.0	
Q1, Q3	45.4, 224.0	40.5, 252.8	45.1, 229.0	
Range	(0.4-2501.0)	(0.4-2430.0)	(0.4-2501.0)	
**Roche U/mL [Elecsys Anti-SARS-CoV-2 S]: High titer**				0.7131[2]
0) <132 U/mL	49 (52.7%)	47 (50.0%)	96 (51.3%)	
1) >=132 U/mL	44 (47.3%)	47 (50.0%)	91 (48.7%)	
**Abbott [SARS-CoV-2 IgG]**				0.2673[1]
Median	4.5	5.5	4.9	
Q1, Q3	2.3, 6.5	2.7, 6.9	2.5, 6.7	
Range	(0.0-8.5)	(0.0-8.2)	(0.0-8.5)	

Summary of 187 samples tested across six assays
By sex

	F (N=93)	M (N=94)	Total (N=187)	p value
**Abbott [SARS-CoV-2 IgG]: High titer**				0.4227[2]
0) Index (S/C)<4.5	47 (50.5%)	42 (44.7%)	89 (47.6%)	
1) Index (S/C)>=4.5	46 (49.5%)	52 (55.3%)	98 (52.4%)	
				0.8500[1]
**GenScript [cPass SARS-CoV-2 Neutralization Antibody]**				
Median	74.9	72.6	74.0	
Q1, Q3	50.5, 85.9	51.5, 84.4	51.0, 85.3	
Range	(9.4-98.0)	(4.2-97.6)	(4.2-98.0)	
**GenScript [cPass SARS-CoV-2 Neutralization Antibody]: High titer**				0.7005[2]
0) Inhibition<68%	37 (39.8%)	40 (42.6%)	77 (41.2%)	
1) Inhibition>=68%	56 (60.2%)	54 (57.4%)	110 (58.8%)	

(report generated on 26APR2021)
[1]Wilcoxon [2]Chi-Square
